# Practice of oxygen use in anesthesiology – a survey of the European Society of Anaesthesiology and Intensive Care

**DOI:** 10.1186/s12871-022-01884-2

**Published:** 2022-11-14

**Authors:** Martin Scharffenberg, Thomas Weiss, Jakob Wittenstein, Katharina Krenn, Magdalena Fleming, Peter Biro, Stefan De Hert, Jan F. A. Hendrickx, Daniela Ionescu, Marcelo Gama de Abreu, for the European Society of Anaesthesiology and Intensive Care

**Affiliations:** 1 Department of Anaesthesiology and Intensive Care Medicine, Pulmonary Engineering Group, University Hospital Carl Gustav Carus, Technische Universität Dresden, Dresden, Germany; 2 Department of Anaesthesia, General Intensive Care and Pain Medicine, Medical University of Vienna, Vienna, Austria; 3 Department of Anesthesiology and Intensive Care, Czerniakowski Hospital, Warsaw, Poland; 4 Institute of Anesthesiology, University Hospital Zurich, Zurich, Switzerland; 5 Department of Anesthesiology and Perioperative Medicine, Ghent University Hospital – Ghent University, Ghent, Belgium; 6 Department of Anesthesiology, OLV Hospital, Aalst, Belgium; 7 Department of Basic and Applied Medical Sciences, Ghent University, Ghent, Belgium; 8 Department of Anesthesiology, UZLeuven, Leuven, Belgium; 9 Department of Cardiovascular Sciences, KULeuven, Leuven, Belgium; 10 Department of Anaesthesia and Intensive Care, Iuliu Hatieganu University of Medicine and Pharmacy, and Clinical Department of Anaesthesia and Intensive Care, Regional Institute for Gastroenterology and Hepatology, Cluj-Napoca, Romania; 11 Department of Intensive Care and Resuscitation, Anesthesiology Institute, Cleveland Clinic, 9500 Euclid Ave, Cleveland, OH 44195 USA; 12 Department of Outcomes Research, Anesthesiology Institute, Cleveland Clinic, 9500 Euclid Ave, Cleveland, OH 44195 USA

**Keywords:** Oxygen therapy, Supplemental oxygen, Patient safety, Inspiratory fraction of oxygen, Perioperative care, Intensive care medicine, Critical emergency medicine, Oxygen toxicity, WHO guidelines on surgical site infection prevention

## Abstract

**Background:**

Oxygen is one of the most commonly used drugs by anesthesiologists. The World Health Organization (WHO) gave recommendations regarding perioperative oxygen administration, but the practice of oxygen use in anesthesia, critical emergency, and intensive care medicine remains unclear.

**Methods:**

We conducted an online survey among members of the European Society of Anaesthesiology and Intensive Care (ESAIC). The questionnaire consisted of 46 queries appraising the perioperative period, emergency medicine and in the intensive care, knowledge about current recommendations by the WHO, oxygen toxicity, and devices for supplemental oxygen therapy.

**Results:**

Seven hundred ninety-eight ESAIC members (2.1% of all ESAIC members) completed the survey. Most respondents were board-certified and worked in hospitals with > 500 beds. The majority affirmed that they do not use specific protocols for oxygen administration. WHO recommendations are unknown to 42% of respondents, known but not followed by 14%, and known and followed by 24% of them. Respondents prefer inspiratory oxygen fraction (FiO_2_) ≥80% during induction and emergence from anesthesia, but intraoperatively < 60% for maintenance, and higher FiO_2_ in patients with diseased than non-diseased lungs. Postoperative oxygen therapy is prescribed more commonly according to peripheral oxygen saturation (SpO_2_), but shortage of devices still limits monitoring. When monitoring is used, SpO_2_ ≤ 95% is often targeted. In critical emergency medicine, oxygen is used frequently in patients aged ≥80 years, or presenting with respiratory distress, chronic obstructive pulmonary disease, myocardial infarction, and stroke. In the intensive care unit, oxygen is mostly targeted at 96%, especially in patients with pulmonary diseases.

**Conclusions:**

The current practice of perioperative oxygen therapy among respondents does not follow WHO recommendations or current evidence, and access to postoperative monitoring devices impairs the individualization of oxygen therapy. Further research and additional teaching about use of oxygen are necessary.

**Supplementary Information:**

The online version contains supplementary material available at 10.1186/s12871-022-01884-2.

## Background

Oxygen therapy is an integral part of patient care in perioperative, emergency, and intensive care medicine. While oxygen can improve the oxidative neutrophilic immune defence, a high inspiratory fraction of oxygen (FiO_2_) favours the formation of reactive oxygen species and triggers the release of inflammatory markers, resulting in cell apoptosis and death [1–4]. In the clinical setting, high FiO_2_ was associated with atelectasis and impaired hypoxic vasoconstriction [5], increased shunt fraction [6], lung injury, and tracheobronchitis [7]. In 2016, the World Health Organisation (WHO) recommended the use of FiO_2_ of 80% during surgery and up to 6 h postoperatively, as a means to reduce the risk of surgical site infections (SSI) [8]. However, recent meta-analyses have suggested that the ability of high FiO_2_ to reduce SSI may either apply to intubated surgical patients only [9], or not be present at all [10]. Furthermore, intraoperative high FiO_2_ has been associated with postoperative atelectasis and reduced pulmonary function [11], but not with impaired clinical outcomes [12]. In critical emergency medicine, supplemental oxygen could prevent ischemic insults. In acute critical situations during anesthesia, e.g., when a technical problem with ventilation occurs, a high oxygen concentration also buys time to solve the problem. However, the effects of oxygen are both dose- and organ-dependent [13–16]. While oxygenation targets in the intensive care unit (ICU) setting have not been adequately defined, the use of high FiO_2_ in the first 24 h after ICU admission has been linearly associated with in-hospital mortality [17]. The lack of consensus, as well as the heterogeneous practice of oxygen use, might compromise patient safety, and expose caregivers to possible legal consequences. Clearly, knowing how oxygen is administered might contribute to reduce risks and improve clinical practice. In view of these facts, we designed and conducted an online survey among members of the European Society of Anaesthesiology and Intensive Care (ESAIC) to gain insight into the current practice of oxygen use in anesthesia, critical emergency, and intensive care medicine.

## Methods

This was a voluntary survey conducted on behalf of the ESAIC Board of Directors (decision from 19th December, 2018), and following a recommendation of the ESAIC Research Committee. Members of the taskforce responsible for planning the survey were selected among ESAIC active members who had experience in the fields and were involved in research. All methods were carried out in accordance with the Declaration of Helsinki as well as relevant guidelines and regulations. Approval of an ethics committee, Internal Review Board, or licensing committee was not necessary because participation did not interfere with the psychological or physical integrity of participants, no biomaterials were obtained, nor could participants be identified by the responses given in this completely anonymous survey [18] (also see Declarations). All subjects consented to participate by submitting the survey, by which informed consent was obtained from all participants. Because all participants were legally competent and decided to participate themselves, the option of consent by legal guardians was not applicable. AirLiquide (Paris, France) provided financial support to conduct the survey, but had no influence on the selection of the task force members, the topics addressed in the survey, analysis of results, or compilation of the manuscript. Taskforce members met on multiple occasions in 2019 by videoconference to discuss the scope of the survey. After obtaining consensus, the survey was implemented using the SurveyMonkey® platform. Cookies were not used to assign unique user identifier to each client computer, and IP address of the client computer was not used to identify potential duplicate entries. Log file analysis for identification of multiple entries was not used. An email with the survey link (https://esaresearch.limequery.com/893287?lang=en) was sent by the ESAIC secretariat to 37,872 ESAIC members. The survey electronic link was also accessible from the ESAIC Facebook page during the period from 31st January to 11th June, 2020. No incentives for participation were offered. The questionnaire consisted of a total of 46 questions, which were subdivided as follows: Nine questions about the respondent and hospital characteristics; 16 questions related to the perioperative period (including surgical wards); seven questions related to critical emergency medicine; 10 questions addressing intensive care; and four questions on toxicity and drug interaction. The survey also included a table with multiple images of commonly used oxygen administration devices with queries aimed at evaluating the familiarity of respondents with equipment and monitoring practice. The survey was designed to be completed in less than 15 minutes, and respondents were allowed to skip questions. Most questions were multiple choice, while some of them allowed multiple answers, while others were adaptive, that is, being conditionally displayed based on responses to other questions. No more than 10 questionnaire items were displayed per page. Respondents were able to review and change their answers through a Back button. Completeness checks were note implemented. Questionnaires that were terminated early, that is, from respondents who did not go through all pages, were also analysed. No timeframe was used as cut-off point for considering questionnaires. The complete questionnaire and the table with devices for oxygen administration are available in the online supplement (see Additional file 1). This survey was exploratory and descriptive in nature. However, we conducted post-hoc analyses for homogeneity of given answers between different categories, e.g., geographical regions or hospital types, using χ^2^ test (SPSS, version 28, IBM, USA). Weighting of items and propensity scores have not been used to adjust for non-representative samples. Significance was accepted at *p* < 0.05 and adjustments for multiple testing were conducted according to Bonferroni.

## Results

Among the 37,872 ESAIC members who received the email, 9517 opened the link to the survey, 999 members at least started the survey, and 798 anesthesiologists, mainly from Europe and the Americas, representing 2.1% of the total number of ESAIC members, responded to the survey (Table 1). Most respondents were board-certified in anesthesiology (*n* = 759; 95.1%), but many of them held additional certifications in intensive care medicine, critical emergency medicine, and pain therapy. The most common double certification was anesthesiology/intensive care medicine (*n* = 379; 47.5%), followed by anesthesiology/critical emergency medicine (*n* = 120; 15.0%), and anesthesiology/pain therapy (*n* = 79; 9.9%). Most respondents worked in university hospitals, heart centres, or another type of tertiary care facility. Hospital size varied from ≥500 beds (*n* = 383; 49.3%), to 100 to 499 beds (*n* = 334; 41.9%), and < 100 beds (*n* = 63; 7.9%). The majority of respondents worked in hospitals with ≤20 operation rooms.

**Table 1:** Characteristics of participants who completed the survey

*Characteristic*	*All respondents n = 798*
*Continent, n (%)*	
Europe	653 (81.8)
Americas	33 (4.1)
Eastern Mediterranean	28 (3.5)
Western Pacific	27 (3.4)
South-East Asia	19 (2.4)
Africa	4 (0.5)
Not informed	34 (4.3)
*Board certification, n (%)*	
Yes	770 (96.5)
No	28 (3.5)
Not informed	0 (0.0)
*Field of board certification, n (%) (multiple answers allowed)*	
Anaesthesiology	795 (99.6)
Intensive Care Medicine	384 (48.1)
Critical Emergency Medicine	123 (15.4)
Pain Therapy	79 (9.9)
Paediatrics	18 (2.3)
Neonatology	10 (1.3)
Internal Medicine - Pneumology	5 (0.6)
Internal Medicine - Cardiology	4 (0.5)
Other	22 (2.8)
Not informed	0 (0.0)
*Field of primary clinical activity, n (%)*	
General anaesthesiology	529 (66.3)
Cardiac anaesthesiology	57 (7.1)
Paediatric/neonatal anaesthesiology	48 (6.0)
Surgical intensive care medicine	41 (5.1)
Medical intensive care medicine	33 (4.1)
Cardio-thoracic intensive care medicine	11 (1.4)
Emergency medicine	9 (1.1)
Critical emergency medicine	6 (0.8)
Pain therapy	4 (0.5)
Neonatal/paediatric intensive care medicine	2 (0.3)
Other	40 (5.0)
Not informed	18 (2.3)
*Type of primary institution, n (%)*	
University hospital	379 (47.5)
General hospital	122 (14.1)
Private hospital	120 (13.9)
Tertiary care hospital (neither university hospital nor heart centre)	106 (12.2)
Secondary care hospital	68 (7.9)
Heart centre	38 (4.4)
Private practice hospital	25 (2.9)
Other	8 (0.9)
Not informed	0 (0.0)
*Total number of beds, n (%)*	
≥ 500	383 (47.9)
100 to 499	334 (41.9)
< 100	63 (7.9)
Not informed	18 (2.3)
*Total number of operation rooms, n (%)*	
< 20	261 (32.7)
20 to 40	180 (22.6)
≥ 40	79 (9.9)
Not informed	278 (34.8)

Sum of percentages may exceed 100% due to more than one possible answer, where applicable

### Oxygen therapy in general

More than 70% (*n* = 559) of respondents stated that they do not use specific protocols or guidelines for oxygen therapy, regardless of geographical location, board certifications status, and types of primary institution (Table 2).

**Table 2:** Oxygen therapy in general practice - Not using specific protocols or guidelines

*Subgroup of respondents*	*Not using protocols or guidelines*	*p*
*Continent, n (%)*		0.104
Europe	476/653 (72.9)	
Americas	29/33 (87.9)	
Eastern Mediterranean	17/28 (60.7)	
Western Pacific	14/27 (51.9)	
South – East Asia	12/19 (63.2)	
Africa	4/4 (100.0)	
*Board certification, n (%)*		0.661
Anaesthesiology	547/759 (72.1)	
Intensive Care Medicine	286/384 (74.5)	
Critical Emergency Medicine	93/123 (75.6)	
*Type of primary institution, n (%)*		0.359
University hospital	257/379 (67.8)	
General hospital	87/122 (71.3)	
Private hospital	84/120 (78.3)	
Tertiary care hospital (neither university hospital nor heart center)	82/106 (77.4)	

*p* Pearson-Chi-Square

### Use of oxygen in the perioperative setting

**Fig. 1:**
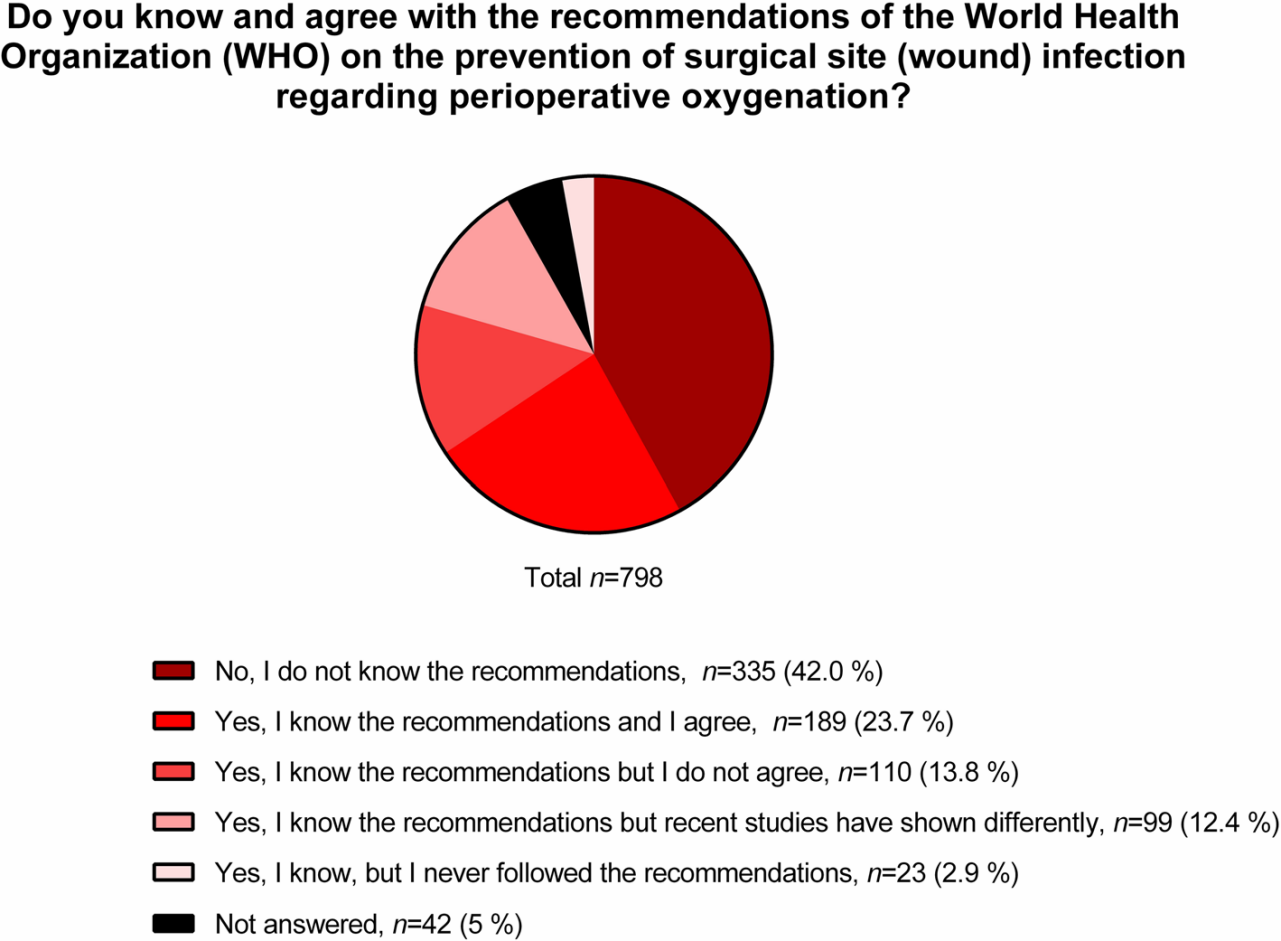
Respondent’s knowledge and acceptance of the recent guidelines of the World Health Organization

As shown in Fig. 1, approximately 42% (*n* = 335) of respondents were not familiar with the current recommendations of the WHO on SSI prevention by perioperative oxygen therapy [8], while 13.8% (*n* = 110) knew but do not agree with them, 2.9% (*n* = 23) have never followed those recommendations, and 12.4% (*n* = 99) claim that recent studies diverge from them. Yet, 23.7% (*n* = 189) of participants knew and agreed with the recommendations.

**Fig. 2:**
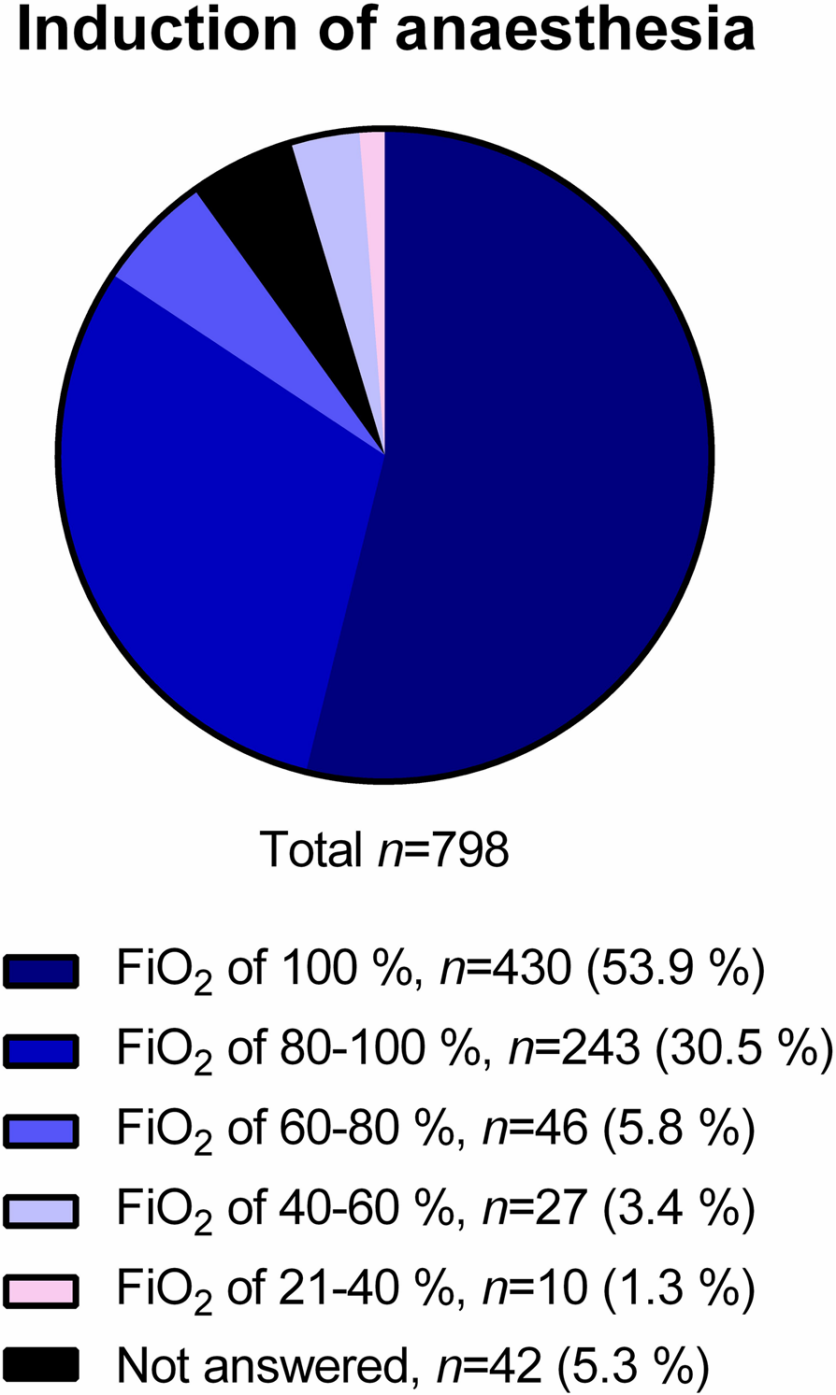
Preferred inspiratory oxygen fraction (FiO_2_) for induction of anaesthesia

For induction of anesthesia, an FiO_2_ of 100% was preferred by the majority of respondents, but approximately one third of participants selected FiO_2_ between 80 and 100% (Fig. 2).

During maintenance of general anesthesia, the majority of respondents (*n* = 379; 47.5%) preferred FiO_2_ of 40–60%, followed by FiO_2_ of 21–40% (*n* = 295; 37.0%), with less than 10% (*n* = 81) of participants using FiO_2_ ≥ 60%.

During the emergence period, FiO_2_ between 80 and 100% was preferred by approximately 35% (*n* = 275) of participants, followed closely by FiO_2_ of 100% (*n* = 268; 33.8%). Analysis of FiO_2_ use for induction, maintenance, and emergence per geographical region is reported in an additional file in more detail (see Additional file 2). For induction of anesthesia, proportions were similarly distributed. During maintenance, FiO_2_ of 100% was selected statistically more often in Africa than in other regions. During emergence, 100% FiO_2_ was selected more often in South-East Asia and less frequently in Europe and Eastern Mediterranean regions.

More than the half of respondents replied also that supplemental oxygen is inconsistently used, that is, only sometimes (*n* = 220; 27.6%) or rarely (*n* = 217; 27.2%), when transferring patients from the operation room to the post-anesthesia care unit (PACU), as well as from the PACU to surgical wards (sometimes, *n* = 290; 36.3%; rarely, *n* = 301; 37.7%). While 37.7% (*n* = 301) claimed they do not distinguish between patients with diseased vs. non-diseased lungs regarding FiO_2_, 30.0% (*n* = 239) of the respondents preferred higher FiO_2_ and 24.2% (*n* = 193) used lower FiO_2_ during anesthesia.

Approximately 56% (*n* = 447) of all respondents recommended oxygen therapy postoperatively in the post-surgery ward depending on actual peripheral oxygen saturation (SpO_2_), while 19.8% (*n* = 158) prescribed this therapy for high-risk patients only, 8.7% (*n* = 69) recommend supplemental oxygen regularly, and 2.6% (*n* = 21) advocated it in patients who required proportionally high doses of opioids. Roughly 2% (*n* = 18) never recommend oxygen in the post-surgery ward.

Yet, 66.7% (*n* = 532) of respondents considered the monitoring of SpO_2_ during administration of oxygen in the ward mandatory, irrespective of risk. However, only 54.5% (*n* = 435) of participants informed that SpO_2_ is monitored regularly in all post-surgery patients, while 27.4% (*n* = 219) reported that their institutions monitor SpO_2_ in high-risk patients only. Among all respondents, 53.6% (*n* = 428) stated that SpO_2_ data is also documented in intervals in patient’s records when oxygen is administered in the post-surgery ward, while only 14.2% (*n* = 113) informed that those data are recorded automatically. Approximately 5% (*n* = 38) stated that their institutions never document SpO_2_ while administering oxygen in post-surgery wards. The main reason for not monitoring SpO_2_ postoperatively on wards was lack of devices (*n* = 389; 48.8%), followed by increased workload for nurses (*n* = 178; 22.3%), which was statistically similar between the analysed geographical regions, as shown in more detail in an additional table (see Additional file 3).

For roughly 31% (*n* = 249) of participants, supplemental oxygen therapy on the surgical ward should be prescribed upon patients’ comorbidities, while 14% (*n* = 112) of those colleagues did not guide oxygen therapy based on SpO_2_ thresholds. Physicians who used SpO_2_ to guide the decision on oxygen therapy more frequently, reported a preferred SpO_2_ threshold of 92% (*n* = 164; 20.6%), followed by 90% (*n* = 100; 12.5%), 95% (*n* = 67; 8.4%), and 85% (*n* = 21; 2.6%).

### Use of oxygen in critical emergency medicine

The majority of respondents (*n* = 540; 67.7%) considered that supplemental oxygen reduces the risk of death in critical emergency medicine, while 9.3% (*n* = 74) claimed the opposite. In critical emergency patients aged ≥80 years, approximately 83% of physicians used supplemental oxygen, i.e. sometimes (*n* = 264; 33.1%), usually (*n* = 233; 29.2%), or almost always (*n* = 167; 20.9%).

Patients who presented with respiratory distress were treated almost always (*n* = 453; 56.8%), usually (*n* = 189; 23.7%), or sometimes (*n* = 40; 5.0%) with supplemental oxygen by participants in this survey.

In patients presenting with chronic obstructive pulmonary disease (COPD), more than 79% (*n* = 632) of respondents would use supplemental oxygen (sometimes, *n* = 343, 43%; usually, *n* = 188, 23.7%; almost always, *n* = 101, 12.66%).

In patients with acute myocardial infarction, supplemental oxygen was used almost always, sometimes, or usually by 42.0% (*n* = 335), 21.7% (*n* = 173), and 14.8% (*n* = 118) of respondents, respectively. In patients with stroke being treated by respondents, a similar pattern to myocardial infarction was reported (almost always, *n* = 260, 32.6%; sometimes, *n* = 199, 24.9%; usually, *n* = 173, 21.7%).

Respondents considered supplemental oxygen significantly less frequently in critical emergency patients presenting with any other than the following conditions ≥80 years old, respiratory distress, COPD, myocardial infarction, and stroke: Rarely, 22.0% (*n* = 151) and never, 3.1% (*n* = 21), whereas only 6.5% (*n* = 52) almost always administer oxygen to these patients (*p* < 0.001 for any other conditions vs. each of the afore-mentioned).

### Use of oxygen in intensive care medicine

Approximately 26% (*n* = 209) of respondents stated they administer supplemental oxygen independently from SpO_2_ in spontaneously breathing ICU patients with healthy lungs, especially in high risk patients, while 51.4% (*n* = 410) supplied oxygen under a certain target SpO_2_. In these patients, SpO_2_ was mainly targeted at 94–96% (*n* = 214; 26.8%), closely followed by 92–94% (*n* = 174; 21.8%), 90–92% (*n* = 105; 13.2%), 97–100% (*n* = 65; 8.2%), and 88–90% (*n* = 31; 3.9%).

**Fig. 3:**
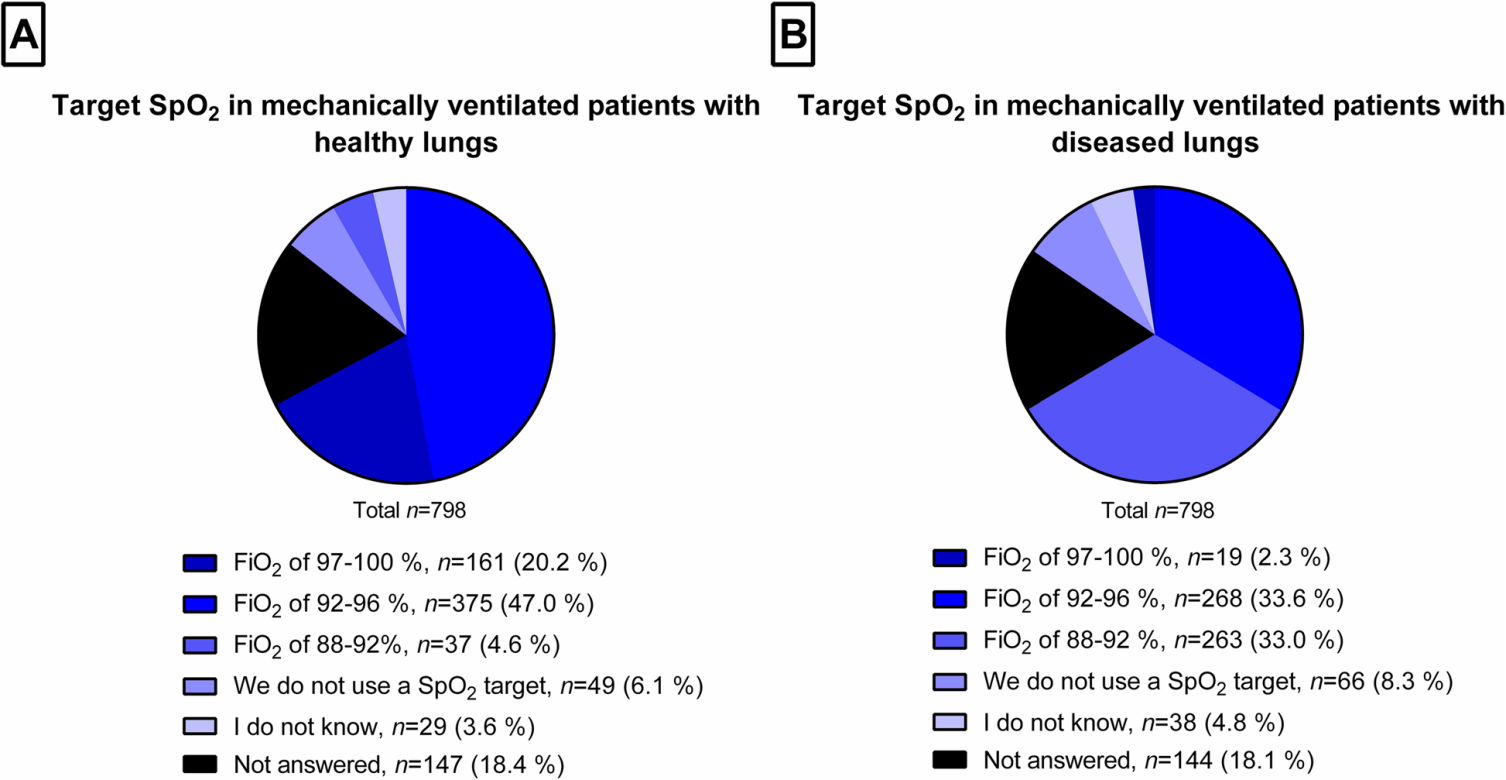
Target SpO_2_ in mechanically ventilated patients with healthy (panel **A**) and diseased lungs (panel **B**)

Figure 3 shows SpO_2_ targets in mechanically ventilated ICU patients. Most participants preferred targeting SpO_2_ at 92–96% in patients with healthy (*n* = 375; 47.0%) as well as non-healthy (*n* = 268; 33.6%) lungs. The second-preferred SpO_2_ target was 97–100% in patients with healthy lungs (*n* = 161; 20.2%), but 88–92% in patients with non-healthy lungs (*n* = 263; 33.0%). Less than 10% of participants reported they do not guide oxygen therapy according to SpO_2_ in this sub-population, independently of lung disease. According to the participants, SpO_2_ targets were reached in their ICUs most of the time (*n* = 462; 57.9%), sometimes (*n* = 133; 16.7%), or always (*n* = 35; 4.4%).

More than half of the respondents (*n* = 441; 55.3%) were concerned about both hyperoxemia and hypoxemia in ICU patients. Nearly 15.0% (*n* = 120) feared rather hypoxemia and 8.8% (*n* = 70) are more afraid of hyperoxemia, whereas 2.9% (*n* = 23) had no major concerns regarding extremes of oxygen therapy. While arterial partial pressure of oxygen (*P*aO_2_) was used to detect hyperoxemia by more than 50% (*n* = 408) of participants, approximately 40% (*n* = 316) would rely on both SpO_2_ and *P*aO_2_ to detect hypoxemia. Yet, most respondents preferred monitoring oxygen treatment with *P*aO_2_ (*n* = 396; 49.6%), followed by SpO_2_ (*n* = 171; 21.4%), and arterial oxygen saturation (SaO_2_) (*n* = 80; 10.0%) in the ICU.

### Awareness about oxygen toxicity

Approximately 55% (*n* = 436) of participants considered that FiO_2_ of 100% applied > 4 h can be toxic to the central nervous system, while 31.3% (*n* = 250) were concerned when using FiO_2_ of 80% for > 8 h. Virtually 35% (*n* = 279) believed that oxygen can be toxic at any concentration higher than normal under atmospheric pressure conditions.

Less than 13% (*n* = 100) of the participants in the survey saw a potential for lung toxicity when FiO_2_ is kept below 50%, but the percentage of physicians concerned about this harmful effect increased to 28.9% (*n* = 231), 48.0% (*n* = 383), and 66.5% (*n* = 531) when FiO_2_ of 60, 80, and 100% were used, respectively.

The main potential side effects of FiO_2_ > 50% in adults that respondents were concerned about were formation of atelectasis (*n* = 564; 70.7%), followed by degradation of lung surfactant (*n* = 419; 52.5%), decline in vital capacity (*n* = 384; 48.1%), and epigastric pain (*n* = 43; 5.4%). Yet, in preterm newborns, retinopathy, retrolental fibroplasia, bronchopulmonary dysplasia, and nephrotoxicity, were identified as possible complications of high oxygen therapy by 57.8% (*n* = 461), 53.5% (*n* = 427), 50.3% (*n* = 401), and 7.5% (*n* = 60) of participants, respectively.

### Devices for administration of oxygen

Table 3 summarises the devices used to administer oxygen, as addressed in the survey. The five most frequently used devices by respondents within the last 4 weeks preceding survey completion were anesthetic facemask (*n* = 615), followed by nasal cannula (*n* = 612), simple facemask/‘Hudson’ mask (*n* = 543), ICU ventilators (*n* = 475), and resuscitation bag with mask (*n* = 453), respectively.

**Table 3:** Devices for oxygen administration used within four weeks prior to completing the survey

*Devices used within last four week prior to responding the survey*	*Respondents, n (%)*
Anaesthetic facemask (with an anaesthetic breathing circuit)	615 (77.1)
Nasal cannula	612 (76.7)
Simple facemask/Hudson mask	543 (68.0)
ICU Ventilator	475 (59.5)
Resuscitation bag and mask	453 (56.8)
Aerosol mask	409 (51.3)
Oxygen rotameter	340 (42.6)
Non-rebreather facemask	335 (42.0)
High flow nasal oxygen	265 (33.2)
Venturi mask	254 (31.8)
Nasal cannula, that delivers oxygen and measures end-tidal CO_2_	254 (31.8)
Nasal mask	180 (22.6)
Nasopharyngeal catheter	123 (15.4)
Face tent	34 (4.3)
Oxygen hood	25 (3.1)

The total number of respondents was 798; sum of percentages exceed 100% due to more than one possible answer

*ICU* Intensive care unit, *CO*_*2*_ Carbon-dioxide

## Discussion

The main findings of this international survey are that: 1) the recent recommendations of the WHO on perioperative oxygen therapy are not followed by most respondents; 2) FiO_2_ ≥ 80% is commonly used during induction and emergence from anesthesia, but FiO_2_ < 60% is preferred for maintenance, whereby higher values are used in patients with diseased compared with non-diseased lungs; 3) postoperative oxygen therapy is prescribed more commonly according to SpO_2_, whereby the lack of devices still limits the broad use of SpO_2_ monitoring; 4) among respondents using monitoring devices, values of 95% and lower are preferred; 5) in critical emergency medicine, supplemental oxygen is used frequently in patients aged ≥80 years, presenting with respiratory distress, COPD, myocardial infarction, and stroke; 6) in the ICU, administration of oxygen is usually guided by SpO_2_, depending on presence or absence of lung diseases, and fear of hypoxemia was greater than hyperoxemia; 7) most respondents are concerned about the toxic effects of oxygen on the lungs in adults, and on eyes, lungs and kidneys in preterm newborns; and finally, 8) the five most commonly used devices for oxygen administration are anesthetic face mask, nasal cannula, simple facemask/‘Hudson’ mask, ICU ventilators, and resuscitation bag with mask.

It is not surprising that most respondents are either not aware, or did not agree with the WHO recommendations on the perioperative use of oxygen [8]. These recommendations have been originally compiled without the participation of anesthesiologists, and their legitimacy has been challenged [19–23]. Furthermore, some of the trials that backed those recommendations came under scrutiny [24–26], and one was retracted [27, 28], which likely contributed to raise scepticism among anesthesiologists about the recommendations. Our results also show that also the recent WHO updated analysis, which excluded questionable trials and included new studies since the WHO guideline review was published [9], does not influence the decision of anesthesiologists regarding perioperative hyperoxia. A possible explanation is that the reduction of SSI by high perioperative FiO_2_ is limited to surgical patients under general anesthesia with tracheal intubation, limiting the acceptance of the analyses. However, we cannot completely rule out the possibility that these recommendations are not yet widespread within the anesthesiology community. The use of high FiO_2_ during induction and emergence from anesthesia by most respondents was not surprising. Although the incidence of unanticipated difficult intubation in the general surgical population is relatively low, ranging between 0.01% [29] and 0.43–0.52% [30], high FiO_2_ prolongs the tolerable apnoea time, that is, the time until SpO_2_ decreases to 90%, to as much as 10 min [31], providing a substantial safety margin. As one can infer from the results, the FiO_2_ was decreased for the duration of anesthesia until extubation once the airway was secured, which might be explained by concerns related to formation of atelectasis and oxygen toxicity. Apparently, those risks outweighed the putative beneficial effects of high intraoperative FiO_2_ against postoperative nausea and vomiting [32], as well as the fear of acutely impaired oxygen transport due to accidental extubation and massive hemorrhage [33]. The use of higher FiO_2_ during extubation likely reflects the fear of desaturation, since this period of anesthesia may be accompanied by impaired ventilation due to laryngospasm [34], residual neuromuscular blockade, opioid induced respiratory depression, and presence of secretions in the airways, as well as intrapulmonary shunt due to atelectasis. This fear is less when patients are transferred to the PACU, or even to the ward, as indicated by a relatively low percentage of participants who use supplemental oxygen in that period. The presence of lung disease in surgical patients does not influence the use of perioperative oxygen likely due to conflicting concerns of worsening lung injury and development of hypoxemia in this subpopulation.

A possible interpretation for the observation that roughly two thirds of respondents recommend monitoring of SpO_2_ on the ward is the concern about patient safety. Also, it might reflect an attempt to individualize the use of supplemental oxygen. In spite of this awareness, SpO_2_ is documented only inconsistently, suggesting that economical constraints might still represent an obstacle to improve patient safety in that period. In fact, pulmonary complications, especially hypoxemia, are relatively common following surgery [35, 36], which is associated with increased need for admission to ICU and prolonged in-hospital length of stay [37]. Different scores and systems were developed to detect patient deterioration early, e.g. the NHS National Early Warning Scores (NEWS, NEWS 2) [38, 39]. Since these scores include non-invasively measured oxygen saturations, not measuring SpO_2_ in the (postsurgical) ward may represent a risk for the patients by making early deterioration detection impossible. Especially in light of the fact that the two most frequently stated reasons for not measuring SpO_2_ were limited availability of devices and increased workload for nurses, our survey reveals an urgent need for action to increase patient safety in this regards. Interestingly, when monitoring is used, SpO_2_ values as low as 90% are selected as threshold for oxygen therapy. Possibly, anesthesiologists do not infer a causal relationship between those SpO_2_ values and outcome measures.

It is worthy of note that more than two thirds of respondents consider the administration of supplemental oxygen to be lifesaving in critical emergency situations, especially in patients aged 80 years and older. Respondents are even more prone to use oxygen therapy in patients presenting with respiratory distress, including those with COPD. Low oxygenation and higher age were predictive of unfavourable outcome in a retrospective analysis [40]. However, a metaanalysis showed that the liberal use of oxygen resulted in increased mortality in critical emergency medicine [13]. It is conceivable that given the conflicting data in the literature, respondents opt for tolerating hyperoxemia in this patient population. In patients with myocardial infarction and stroke, respondents also opt for a liberal approach. This choice is not backed by a metanalysis showing that supplemental oxygen has no beneficial effects on mortality, troponin levels, infarction size, and pain [41]. In non-hypoxic patients with ischemic stroke, continuous low-dose oxygen supplementation during first 72 h after the primary event, or during the night only, did not reduce mortality or disability as compared with control, when oxygen was not administered [42].

The observation that the use of oxygen in spontaneously breathing ICU patients is mostly targeted at SpO_2_ might be explained by the fact that oxygenation is routinely monitored on ICU. Thresholds are between 88 and 94%, supporting the interpretation that whenever monitoring is available, a restrictive approach to supplemental oxygen is preferred by respondents. The shift towards higher SpO_2_ thresholds in mechanically ventilated patients with healthy lungs might reflect an attempt to avoid hypoxemic episodes in those patients. This in contrast with a more restrictive approach in patients with lung diseases, in which lower SpO_2_ thresholds are used, and could be explained by concerns related to oxygen toxicity in previously injured lungs. Yet, respondents seem to be more concerned about extremes of oxygenation in intensive care patients, and prefer blood gas analyses over non-invasive oxygenation monitoring for detection of those events. This preference might be due to the lack of ability of SpO_2_ to detect extremes of oxygenation, and is in agreement with the results of a narrative review [43].

The survey revealed that respondents are aware of the potential toxic effects of oxygenation. Surprisingly, approximately one third see a potential for harm at any concentration higher than atmospheric, especially in the lungs of adults, and in different organ systems in preterm newborns.

Although not specifically designed to elucidate the different application forms of oxygen, this survey showed that conventional, very common interfaces, i.e. the anesthesia facemask, nasal cannula, and simple facemask, are more frequently used for oxygen therapy than other devices addressed. This likely reflects the fact that most respondents have a background in anesthesiology and work in perioperative care.

### Potential implications of this survey

The results indicate that educational efforts are needed to improve the use of oxygen therapy. Available evidence might be also used by manufacturers of devices, which could suggest adequate thresholds for oxygenation, and even be combined in form of closed loop systems to adapt therapy according to individual needs. Importantly, the survey suggests that it is necessary to increase the availability of devices for non-invasive monitoring of oxygenation, especially postoperatively on surgical wards, given that those measures have the potential to increase patient safety.

### Limitations

This survey had several limitations. First, measured against the actual number of ESAIC members, the response rate was relatively low, which can limit the extrapolation of the responses to the entire anesthesiology community. Yet, different geographic areas were represented, and the participation rate did not differ substantially from other surveys of the ESAIC. When we opted for not increasing the response rate by limiting the number of addressees, we likely increased the representability of the results. In fact, results in small subgroups of participants may not adequately reflect general practice any, thus, may not be representative, as for example in Africa, where only four anesthesiologists participated in the survey. However, the majority of respondents worked in Europe, as expected when addressing members of a European scientific society, allowing to draw conclusions in this largest sub-cohort. Furthermore, the total number of participants, at almost 800 respondents, is still quite considerable. Second, the survey was extensive, requiring more than 12 minutes to be completed, which might have impaired attention paid to the last sections due to fatigue. Third, the survey was designed and conducted before the outbreak of the new corona virus disease in 2019. Thus, the results cannot be extrapolated to those patients. However, the survey reveals a considerable heterogeneity in perioperative oxygen use already in pre-pandemic times.

## Conclusions

The current practice of oxygen therapy by physicians completing the survey does not follow recent recommendations of the WHO, and is not always evidence-based. While the risk of hyperoxemia is a concern, hypoxemia is more often feared in the different fields of anesthesiology. Respondents consider oxygenation targets relevant, but limited availability of monitors impairs individualization of oxygen therapy. Further research and additional teaching about oxygen therapy, as well as better access to monitoring are required.

## Supplementary Information

**Additional file 1: Supplementary Material 1.** Complete set of Questions – Oxygen survey.

**Additional file 2: Supplementary Table 1.** FiO_2_ during induction, maintenance, and emergence from anaesthesia by geographical regions.

**Additional file 3: Supplementary Table 2.** Post-surgical oxygen management by geographical regions.

## Data Availability

The datasets used and/or analysed during the current study are available from the corresponding author on reasonable request.

## Abbreviations

COPD: Chronic obstructive pulmonary disease
ESAIC: European Society of Anaesthesiology and Intensive Care
F_i_O_2_: Inspiratory fraction of oxygen
ICU: Intensive care unit
PACU: Post-anesthesia care unit
PaO_2_: Arterial partial pressure of oxygen
SaO2: Arterial oxygen saturation
SpO_2_: Peripheral oxygen saturation
SSI: Surgical site infections
WHO: World Health Organization
