# Supplementary material for: Practice of oxygen use in anesthesiology – a survey of the European Society of Anaesthesiology and Intensive Care
Source: BMC Anesthesiol. 2022 Nov 14;22:350. doi: 10.1186/s12871-022-01884-2 (PMC9660141; doi:10.1186/s12871-022-01884-2)

**Practice of oxygen use in Anaesthesiology – An international survey of the European Society of Anaesthesiology and Intensive Care**

M. Scharffenberg, T. Weiss, J. Wittenstein, K. Krenn, M. Fleming, P. Biro, S. De Hert, J. F. A. Hendrickx, D. Ionescu, and M. Gama de Abreu for the European Society of Anaesthesiology and Intensive Care

**Additional File 1**

**Supplementary Material 1**

**­­Complete set of Questions – Oxygen survey**

**Final draft from 02.01.2020**

General questions:

1. In which **country** do you work?
2. For which **fields** do you hold a board certification? (More than one answer possible)
   1. Anesthesiology
   2. Intensive Care Medicine
   3. Critical Emergency Medicine
   4. Pain Therapy
   5. Internal Medicine – pneumology
   6. Internal Medicine – cardiology
   7. Pediatrics
   8. Neonatology
   9. Other:
3. Please indicate your field of **primary clinical activity**:
   1. General anesthesiology
   2. Cardiac anesthesiology
   3. Pediatric/neonatal anesthesiology
   4. Critical Emergency Medicine
   5. Pain Therapy
   6. Surgical intensive care medicine
   7. Cardiothoracic intensive care medicine
   8. Neonatal/pediatric intensive care medicine
   9. Medical intensive care medicine
   10. Emergency medicine
   11. Other:
4. Please indicate the **type of institution** you are affiliated with (multiple answers possible):
   1. University hospital
   2. Heart center
   3. Tertiary care hospital (neither university hospital nor heart center)
   4. Secondary care hospital
   5. Public hospital
   6. Private hospital
   7. General hospital
   8. Private practice hospital
   9. Other (please specify)
5. What is the **total** **number of beds** of your hospital?
6. What is the **total number of operation rooms** in your hospital?
7. In average, to how many patients do you administer supplementary oxygen in a month?
8. In average, to how many patients do you administer supplementary oxygen in a month in the following places? (note: a given patient can receive supplementary oxygen in different places, and thus counted multiple times)
   1. Operation room (OR)
   2. Intensive care unit (ICU)
   3. Postanesthesia care unit (PACU)
   4. Post surgery ward (surgical or not)
9. Does your department use protocols or guidelines for oxygen therapy?
   1. Yes
   2. No

*Questions that allow more than one answer are marked as such*

**1) Use of oxygen in the perioperative setting**

Which inspiratory oxygen concentration do you most often use during **induction** of general anesthesia?

- 100%
- 80-100%
- 60-80%
- 40-60%
- 21-40%

Which inspiratory oxygen concentration do you most often use during **maintenance** of general anesthesia for surgery?

- 100%
- 80-100%
- 60-80%
- 40-60%
- 21-40%

Which inspiratory oxygen concentration do you most often use during **emergence** from general anesthesia?

- 100%
- 80-100%
- 60-80%
- 40-60%
- 21-40%

Do you administer supplemental oxygen during **transfer** from **the Operation Room (OR) to Postanesthesia Care Unit (PACU)** ?

- Yes, almost always
- Yes, usually
- Yes, sometimes
- No, rarely
- No, never

Do you prescribe supplemental oxygen when patients are transferred **from PACU to the post surgery ward ?**

- Yes, almost always
- Yes, usually
- Yes, sometimes
- No, rarely
- No, never

Do you administer supplemental oxygen in spontaneously breathing patients undergoing surgery? (Caesarean section not included)

- Yes, almost always
- Yes, usually
- Yes, sometimes
- No, rarely
- No, never

Do you administer supplemental oxygen in spontaneously breathing patients undergoing Caesarean section?

- Yes, almost always
- Yes, usually
- Yes, sometimes
- No, rarely
- No, never

Do you know and agree with the recommendations of the World Health Organization (WHO) on the prevention of surgical site (wound) infection regarding perioperative oxygenation?

- yes, I know the recommendations and I agree
- no, I do not know the recommendations
- yes, I know the recommendations but I do not agree
- yes, I know the recommendations but recent studies have shown differently
- yes I know, but I never followed the recommendations

**2) Intraoperative oxygen administration in patients with healthy versus diseased lungs**

Compared with patients with healthy lungs, your general approach in patients with diseased lungs undergoing general anesthesia for surgery is to:

- use higher oxygen fractions due to higher risk of hypoxemia
- use lower oxygen fractions due to higher risk of lung injury
- not distinguish between them regarding use of oxygen fraction

**3) Oxygen use in surgical post surgery wards**

In your hospital, is the SpO_2_ (peripheral capillary oxygen saturation) monitored in the post surgery ward?

- regularly
- in high risk patients with multiple co-morbidities
- in patients requiring high doses of opioids for postoperative pain
- never

If the SpO_2_ is monitored postoperatively on the post surgery ward in your hospital, are the data registered in patients' records?

- never
- regularly, at certain time intervals (e.g. 4h)
- data are registered automatically
- when the SpO_2_ drops below certain limits
- in high risk patients

Do you recommend oxygen therapy postoperatively on the post surgery ward?

- regularly
- in high risk patients
- depending on the SpO_2_
- in patients requiring high doses of opioids
- never

Do you have a SpO_2_ threshold/protocol for supplemental oxygen on the post surgery ward?

- 85%
- 90%
- 92%
- 95%
- none
- depends on patient's co-morbidities

Should monitoring of the SpO_2_ be mandatory during administration of supplemental oxygen?

- yes, always
- yes, but only in high risk patients
- yes, but only in the first 24h postoperatively
- I don't know if it is necessary
- no

If your answer was “**no**” to the previous question, why do think that monitoring of the SpO_2_ is not necessary?

- it does not change outcome
- it does not influence the need for admission in ICU
- it would disturb patients
- it would impair patient mobilization
- other:

What is the main reason for the lack of monitoring of the SpO_2_ in the post surgery ward in your hospital?

*(more than one answer possible)*

- not useful
- not enough monitors/ pulse oximeters
- does not change outcome
- extra work for nurses
- electronic recording not available
- not applicable - the SpO_2_ is continuously monitored in the post surgery ward

**4) Oxygen use in Critical Emergency Medicine**

In your opinion, does supplemental oxygen reduce the risk of death in Critical Emergency Medicine?

- Yes, definitely
- Yes, probably
- No

if you answered “no”, do you think that supplemental oxygen?

- - - does not cause harm
    - might cause harm
- I do not know

Do you administer supplemental oxygen to elderly patients (80 years and older) in Critical Emergency Medicine?

- Yes, almost always
- Yes, usually
- Yes, sometimes
- No, rarely
- No, never

Do you administer supplemental oxygen to patients who present with respiratory distress in Critical Emergency Medicine?

- Yes, almost always
- Yes, usually
- Yes, sometimes
- No, rarely
- No, never

Do you administer supplemental oxygen to patients with Chronic Obstructive Pulmonary Disease (COPD) in Critical Emergency Medicine?

- Yes, almost always
- Yes, usually
- Yes, sometimes
- No, rarely
- No, never

Do you administer supplemental oxygen to patients with myocardial infarction in Critical Emergency Medicine?

- Yes, almost always
- Yes, usually
- Yes, sometimes
- No, rarely
- No, never

Do you administer supplemental oxygen to patients with stroke in Critical Emergency Medicine?

- Yes, almost always
- Yes, usually
- Yes, sometimes
- No, rarely
- No, never

Do you administer supplemental oxygen to patients without any of the above mentioned clinical conditions in Critical Emergency Medicine?

- Yes, almost always
- Yes, usually
- Yes, sometimes
- No, rarely
- No, never

**5) Oxygen in the ICU (Intensive Care Unit)**

Do you use supplemental oxygen in patients with healthy lungs breathing spontaneously in the ICU?

- never
- regularly/in all patients
- in high risk patients (cardiac, anemic, etc.)
- under a certain target SpO_2_

What is the SpO_2_ that you target in patients with healthy lungs breathing spontaneously in the ICU?

- 97-100%
- 94-96%
- 92-94%
- 90-92%
- 88-90%
- none, it depends on the patient

In your opinion, conservative oxygen therapy in the ICU, that is, targeting the minimal acceptable SpO_2_ …

*(more than one answer possible)*

- decreases mortality
- decreases infection rate
- may decrease the incidence of liver failure
- decreases the time on mechanical ventilation
- may decrease the incidence of respiratory failure
- improves outcome after cardiac arrest

What is the target SpO_2_ in mechanically ventilated patients with healthy lungs in the ICU?

- 97-100%
- 92-96%
- 88-92%
- do not know
- we do not use a SpO_2_ target

What is the target SpO_2_ in mechanically ventilated patients with non healthy lungs in the ICU (Intensive Care Unit)?

- 97-100%
- 92-96%
- 88-92%
- do not know
- we do not use a SpO_2_ target

Which of the following variables do you prefer for monitoring oxygen therapy in the ICU?

- SpO_2_
- SaO_2_ (arterial oxyhemoglobin saturation)
- PaO_2_ (arterial partial pressure of oxygen)

Are SpO_2_ targets reached in the ICU (Intensive Care Unit)?

- yes, always
- yes, most of the time
- yes, sometimes
- no, there are always some differences

- If targets are NOT reached,
  - in what cases is this?
  - Why are they not reached?

What is your main concern during oxygen therapy?

- Hyperoxemia (PaO_2_ above the normal range)
- Hypoxemia (PaO_2_ below the normal range)
- Both
- I have no concerns

Which variable do you most often use to screen hyperoxemia?

- PaO_2_
- SpO_2_
- Both
- I do not screen hyperoxemia

Which variable do you most often use to screen hypoxemia?

- PaO_2_
- SpO_2_
- Both
- I do not screen hypoxemia

**6) Toxicity of and drug interactions with oxygen**

In your opinion, in which conditions does central nervous system toxicity of oxygen occur?

*(more than one answer is possible)*

- ventilation with 100% if prolonged for >4 h
- ventilation with 80% if prolonged for >8 h
- by applying high oxygen concentrations above atmospheric pressure

In your opinion, in which conditions does lung toxicity by oxygen occur?

*(more than one answer is possible)*

- prolonged ventilation with 100%
- prolonged ventilation with 80%
- prolonged ventilation with 60%
- prolonged ventilation with 50%
- prolonged ventilation with 35%

Which of the following do you consider to be side effects of high oxygen concentrations (>50%)?

*(more than one answer is possible)*

- decline in lung function (decreasing vital capacity)
- formation of atelectasis
- degradation of surfactant
- epigastric pain at the diaphragm

In preterm newborns, which of the following are complications of high oxygen concentrations ?

*(more than one answer possible)*

- retrolental fibroplasia
- retinopathy
- bronchopulmonary dysplasia
- nephrotoxicity

**6) Devices for oxygen administration**

please change measure to monitor


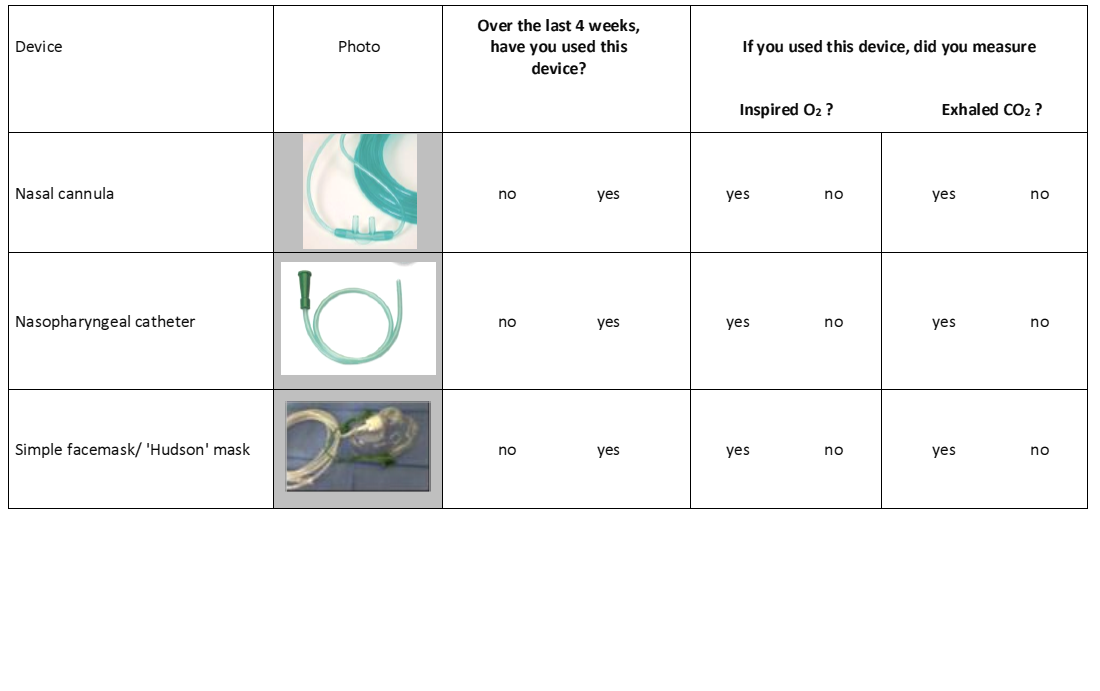


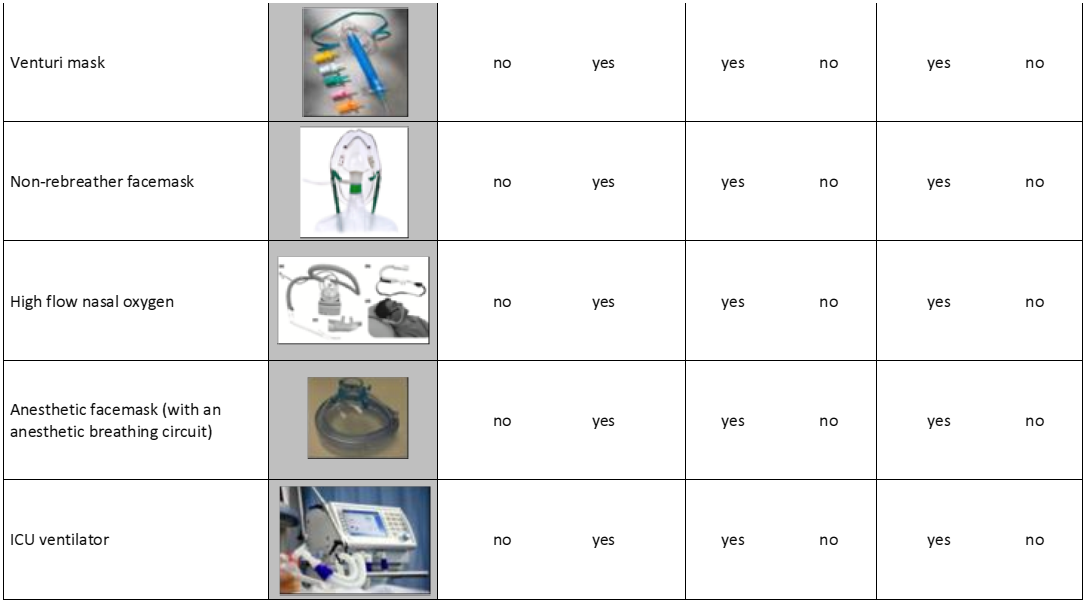


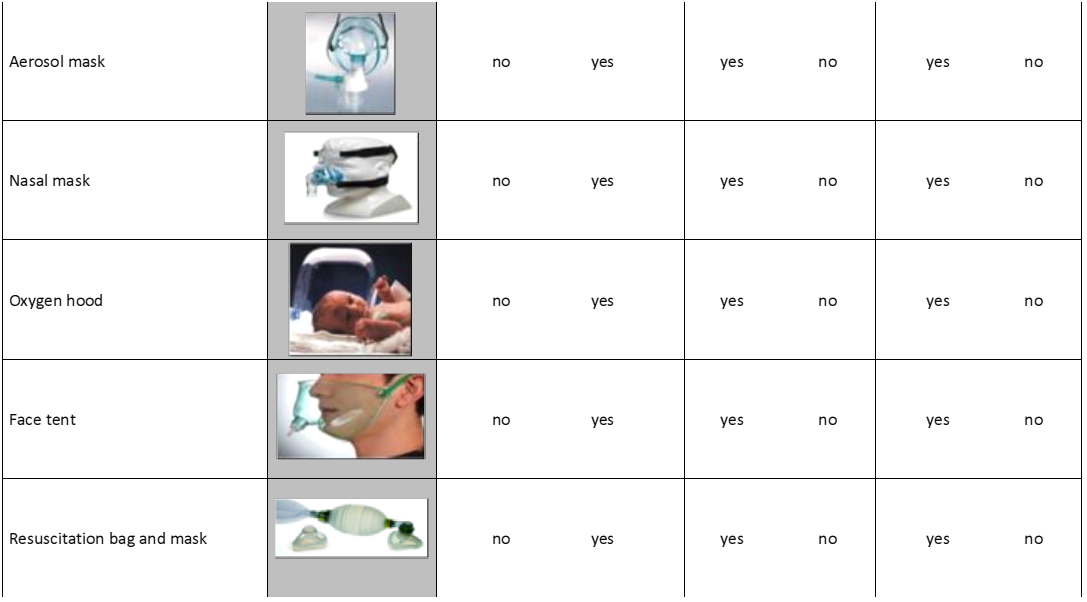

Supplement: Supplementary file 1 — Additional file 1: Supplementary Material 1. Complete set of Questions – Oxygen survey. [file 12871_2022_1884_MOESM1_ESM.docx]
