# Supplementary material for: Practice of oxygen use in anesthesiology – a survey of the European Society of Anaesthesiology and Intensive Care
Source: BMC Anesthesiol. 2022 Nov 14;22:350. doi: 10.1186/s12871-022-01884-2 (PMC9660141; doi:10.1186/s12871-022-01884-2)
Supplement: Supplementary file 2 — Additional file 2: Supplementary Table 1. FiO2 during induction, maintenance, and emergence from anaesthesia by geographical regions. [file 12871_2022_1884_MOESM2_ESM.docx]

**Practice of oxygen use in Anaesthesiology – An international survey of the European Society of Anaesthesiology and Intensive Care**

M. Scharffenberg, T. Weiss, J. Wittenstein, K. Krenn, M. Fleming, P. Biro, S. De Hert, J. F. A. Hendrickx, D. Ionescu, and M. Gama de Abreu for the European Society of Anaesthesiology and Intensive Care

**Additional File 2**

**Supplementary Table 1:** FiO_2_ during induction, maintenance, and emergence from anaesthesia by geographical regions

|  | FiO_2_ | Europe  (*n*=653)  *n* (%) | Eastern Mediterranean  (*n*=28)  *n* (%) | Americas  (*n*=33)  *n* (%) | Africa  (*n*=4)  *n* (%) | Western Pacific  (*n*=27)  *n* (%) | South-East Asia  (*n*=19)  *n* (%) | p |
| --- | --- | --- | --- | --- | --- | --- | --- | --- |
| FiO_2_ during induction from anaesthesia | 100 % | 343 (53) | 19 (68) | 26 (79) | 3 (75) | 15 (56) | 14 (74) | 0.340 |
|  | 80-100 % | 221 (34) | 4 (14) | 5 (15) | 1 (25) | 7 (26) | 2 (11) |  |
|  | 60-80 % | 38 (6) | 2 (7) | 0 (0) | 0 (0) | 2 (7) | 1 (5) |  |
|  | 40-60 % | 22 (3) | 2 (7) | 1 (3) | 0 (0) | 1 (4) | 0 (0) |  |
|  | 21-40 % | 0 (0) | 1 (4) | 0 (0) | 0 (0) | 0 (0) | 1 (5) |  |
|  | No answer | 21 (3) | 0 (0) | 1 (3) | 0 (0) | 2 (7) | 1 (5) |  |
| FiO_2_ during maintenance from anaesthesia | 100 % | 6 (1) | 2 (7) | 2 (6) | 1 (25) **#** | 0 (0) | 0 (0) | **0.019** |
|  | 80-100 % | 16 (3) | 1 (4) | 1 (3) | 0 (0) | 0 (0) | 0 (0) |  |
|  | 60-80 % | 0 (0) | 2 (7) | 1 (3) | 0 (0) | 3 (11) | 0 (0) |  |
|  | 40-60 % | 311 (48) | 16 (57) | 20 (61) | 1 (25) | 12 (44) | 12 (63) |  |
|  | 21-40 % | 254 (39) | 7 (25) | 8 (24) | 2 (50) | 10 (37) | 6 (32) |  |
|  | No answer | 22 (3) | 0 (0) | 1 (3) | 0 (0) | 2 (7) | 1 (5) |  |
| FiO_2_ during emergence from anaesthesia | 100 % | 204 (31) **§** | 21 (75) **§** | 11 (33) | 1 (25) | 9 (33) | 15 (79) **#** | **0.001** |
|  | 80-100 % | 244 (37) | 3 (11) | 9 (27) | 2 (50) | 10 (37) | 2 (11) |  |
|  | 60-80 % | 94 (14) | 3 (11) | 4 (12) | 0 (0) | 4 (15) | 1 (5) |  |
|  | 40-60 % | 58 (9) | 1 (4) | 3 (9) | 1 (25) | 2 (7) | 0 (0) |  |
|  | 21-40 % | 27 (4) | 0 (0) | 4 (12) | 0 (0) | 0 (0) | 0 (0) |  |
|  | No answer | 26 (4) | 0 (0) | 2 (6) | 0 (0) | 2 (7) | 1 (5) |  |

FiO_2_, inspiratory fraction of oxygen; *P*, Pearson-Chi-Square; #, According to post-hoc test reported significantly more often (*P*<0.001); §, According to post-hoc test reported significantly less often (*P*<0.001)
