# Supplementary material for: Practice of oxygen use in anesthesiology – a survey of the European Society of Anaesthesiology and Intensive Care
Source: BMC Anesthesiol. 2022 Nov 14;22:350. doi: 10.1186/s12871-022-01884-2 (PMC9660141; doi:10.1186/s12871-022-01884-2)
Supplement: Supplementary file 3 — Additional file 3: Supplementary Table 2. Post-surgical oxygen management by geographical regions. [file 12871_2022_1884_MOESM3_ESM.docx]

**Practice of oxygen use in Anaesthesiology – An international survey of the European Society of Anaesthesiology and Intensive Care**

M. Scharffenberg, T. Weiss, J. Wittenstein, K. Krenn, M. Fleming, P. Biro, S. De Hert, J. F. A. Hendrickx, D. Ionescu, and M. Gama de Abreu for the European Society of Anaesthesiology and Intensive Care

**Additional File 3**

**Supplementary Table 2:** Post-surgical oxygen management by geographical regions

|  |  | Europe  *n* (%) | Eastern Mediterranean  *n* (%) | Americas  *n* (%) | Africa  *n* (%) | Western Pacific  *n* (%) | South-East Asia  *n* (%) | p |
| --- | --- | --- | --- | --- | --- | --- | --- | --- |
| Total respondents from region |  | *n*=653 | *n*=28 | *n*=33 | *n*=4 | *n*=27 | *n*=19 | *--* |
| Given answers  (multiple answers allowed) |  | *n*=866* | *n*=33 | *n*=46 | *n*=4 | *n*=33 | *n*=21 | *--* |
| Main reason for not monitoring SpO_2_ in the post-surgical ward  (multiple answers allowed) | Not applicable - SpO_2_ is monitored | 145 (17) | 6 (18) | 7 (15) | 0 (0) | 7 (21) | 8 (38) | 0.431 |
|  | Not enough monitors/pulse oximeters | 335 (39) | 8 (24) | 18 (39) | 3 (75) | 10 (30) | 8 (38) |  |
|  | Electronic recording not available | 101 (12) | 8 (18) | 4 (9) | 0 (0) | 3 (9) | 1 (5) |  |
|  | Extra work for nurses | 152 (18) | 7 (21) | 9 (20) | 1 (25) | 4 (12) | 2 (10) |  |
|  | Does not change outcome | 21 (2) | 2 (6) | 1 (2) | 0 (0) | 2 (6) | 1 (5) |  |
|  | Not useful | 21 (2) | 0 (0) | 3 (7) | 0 (0) | 1 (3) | 0 (0) |  |
|  | None of the above | 40 (5) | 4 (12) | 1 (2) | 0 (0) | 2 (6) | 0 (0) |  |
|  | No answer | 51 (6) | 0 (0) | 3 (7) | 0 (0) | 4 (12) | 1 (5) |  |

SpO_2_, peripheral oxygen saturation; *P*, Pearson-Chi-Square; * number of answers exceeds total number of participants due to multiple possible answers
